# IL-15/IL-15Rα in SJS/TEN: Relevant Expression of *IL15* and *IL15RA* in Affected Skin

**DOI:** 10.3390/biomedicines10081868

**Published:** 2022-08-02

**Authors:** Teresa Bellón, Olga González-Valle, Elena Sendagorta, Victoria Lerma, Javier Martínez del Río, Celia Martínez, Guillermo Servera, Carlos González-Herrada, Lucía Cachafeiro, José A. Lorente, Rosario Cabañas, Pedro Herranz, Francisco de Abajo

**Affiliations:** 1 Drug Hypersensitivity Laboratory, Institute for Health Research Hospital Universitario La Paz (IdiPaz), 28046 Madrid, Spain; javier.martinez@cbm.csic.es (J.M.d.R.); celia21955@gmail.com (C.M.); 2 Dermatology Department, Hospital Universitario de Getafe, 28905 Getafe, Spain; ogonzalez@salud.madrid.org (O.G.-V.); carlosmiguel.gonzalez@aedv.es (C.G.-H.); 3 Dermatology Department, Hospital Universitario La Paz-Carlos III-Cantoblanco, IdiPAZ, School of Medicine, Autonomous University of Madrid, 28049 Madrid, Spain; elenasendagorta@hotmail.com (E.S.); gsevera@me.com (G.S.); pherranzp@gmail.com (P.H.); 4 Clinical Pharmacology Unit, Hospital Universitario Príncipe de Asturias, 28805 Alcalá de Henares, Spain; victoria.pielenred@gmail.com (V.L.); francisco.abajo@uah.es (F.d.A.); 5 Department of Biomedical Sciences, University of Alcalá (IRYCIS), 28801 Alcalá de Henares, Spain; 6 Department of Intensive Care Medicine, Hospital Universitario La Paz, 28046 Madrid, Spain; luciacachafeiro@yahoo.es; 7 Department of Intensive Care Medicine, Hospital Universitario de Getafe, 28905 Getafe, Spain; joseangel.lorente@salud.madrid.org; 8 CIBER de Enfermedades Respiratorias, ISCIII, 28029 Madrid, Spain; 9 Departamento de Medicina, Universidad Europea de Madrid, 28670 Madrid, Spain; 10 Bioengineering Department, Universidad Carlos III, 28911 Madrid, Spain; 11 Allergy Department, Hospital Universitario La Paz-Carlos III-Cantoblanco, 28046 Madrid, Spain; charo.cabanas@gmail.com

**Keywords:** Stevens–Johnson syndrome, toxic epidermal necrolysis, Il-15, IL-15Rα

## Abstract

Stevens–Johnson syndrome/toxic epidermal necrolysis (SJS/TEN) is a life-threatening hypersensitivity reaction to medications characterized by keratinocyte apoptosis and skin detachment. IL-15 serum levels have been associated with severity and prognosis of SJS/TEN. We have measured IL-15 concentrations in serum and blister fluid (BF) from patients with SJS/TEN by ELISA and used quantitative RT-PCR to analyze the expression of *IL15* and *IL15RA* (encoding for IL-15 Receptor-α chain) genes in peripheral blood and BF cells, including isolated monocytes, and in affected skin. A positive correlation was found between IL-15 serum levels and a percent of detached skin. BF concentrations were higher, but no correlation was found. Higher *IL15* and *IL15RA* gene expression levels were found in skin-infiltrating blister fluid cells compared to peripheral mononuclear cells. Moreover, *IL15RA* transcripts were barely detected in healthy skin, being the highest expression levels found in samples from two SJS/TEN patients who did not survive. The cutaneous expression of IL-15Rα in SJS/TEN may provide an explanation to the tissue-specific immune cytotoxic response in this clinical entity, and the results suggest that the effects of IL-15 in SJS/TEN patients may be dependent on the expression of its private receptor IL-15Rα in affected skin.

## 1. Introduction

Stevens–Johnson syndrome (SJS) and toxic epidermal necrolysis (TEN) are serious mucocutaneous diseases characterized by full thickness epidermal necrosis leading to skin detachment. Both are severity variants of the same blistering disease only differing in the percentage of body surface area (BSA) affected, with less than 10% of BSA detached in SJS, >30% in TEN, and 10–30% in SJS-TEN overlap cases [1]. SJS/TEN will be used to refer to the whole spectrum of the disease.

SJS/TEN is the most severe cutaneous adverse reaction (SCAR) induced by medications [2]. Although it is a rare disease with an estimated incidence of 1–2 cases per 10^6^ inhabitants/year [3], the high mortality and long-term disabling sequelae highlight its clinical relevance [2,4]. An immune-mediated adverse drug reaction is most often the etiological cause [5,6], aromatic anticonvulsants, allopurinol, sulfonamides, nonsteroidal anti-inflammatory drugs (NSAIDs) and antibiotics being the most frequently associated drugs [7]. However, other etiologic factors may be responsible in up to 15% of cases [8].

A type IVc T cell-mediated hypersensitivity reaction is the pathomechamism underlying SJS/TEN [9]. Although a scarce mononuclear cell infiltrate is typically described in skin biopsies, the longitudinal examination of blister fluid cells (BFC) reveals the presence of considerable amounts of lymphocytes in early samples with increased frequencies of monocytes in late blisters [10,11,12]. Cytotoxic T lymphocytes (CTL) with a memory phenotype and effector functions are found in blister fluids and are thought to be the main mediators of the disease [10,13]. In addition, other cytotoxic leukocytes such as NK cells were identified within blister fluids and could also contribute to keratinocyte killing through the release of cytolytic proteins such as perforin, granzymes and granulysin, which could play a major role in keratinocyte death in SJS/TEN [14,15,16].

IL-15 is a pleiotropic cytokine widely expressed in multiple tissues by different cell lineages, including immune cells (monocytes, macrophages, dendritic cells) and non-immune cells such as fibroblasts and epidermal keratinocytes [17]. It is fundamental for the differentiation and maturation of NK cells and for the acquisition of cytotoxic effector functions by CD8 + CTLs [17], with the expression of granulysin also being highly dependent on IL-15 [18]. It can also work as a chemokine for the recruitment of cytotoxic lymphocytes [17,19].

IL-15 signaling involves the participation of a heterotrimeric receptor complex composed of a common gamma chain (γc) subunit (CD132), shared with other cytokines (IL-2, IL4, IL-7, IL9, IL-21), a β chain subunit shared with IL-2 receptor IL-2/IL-15Rβ (CD122), and a private IL-15-specific α subunit IL-15Rα (CD215). Although it does not play a direct role in signaling, IL-15Rα is a decisive component of the IL-15 receptor complex. IL-15Rα has very high affinity for IL-15 and facilitates the trafficking of IL-15 through the cytoplasm and presentation of IL-15/IL-15Rα complexes on the cell surface. Under inflammatory conditions, IL-15/IL-15Rα can also be cleaved as a complex into the extracellular space. Consequently, IL-15 may exist in three functional forms: soluble, soluble complexes and membrane-bound complexes, which are thought to be the most efficient form of stimulation for T cells or NK cells expressing the heterodimer IL-15Rβ/γc, a mechanism termed ″transpresentation″ [20]. The receptors IL-15Rβ and γc, which confer IL-15 responsiveness to the receiving cells, are present on many hematopoietic cells; however, IL-15Rβ (CD122) expression is highest on CD8 T cells and NK cells. In contrast to the selective expression of IL-15Rβ, IL-15Rα and the cytokine itself are widely expressed by many cell types, although the highest expression is found in monomyeloid cells (monocytes, dendritic cells and macrophages), while it is hardly found in T cells reviewed in [17,21,22,23].

A recently published study found significantly higher levels of several cytokines in the serum of patients with SJS/TEN when compared with patients suffering from DRESS or drug-induced mild exanthematic reactions. Among them, circulating IL-15 serum levels at admission were found to be related to the severity of illness and mortality [24]. IL-15 was thus proposed as a biomarker, and as a putative therapeutic target in SJS/TEN [25]. However, the presence of the cytokine has not been assessed in blister fluids or affected skin, the main target organ of the disease.

In this paper, we have analyzed serum IL-15 levels in a series of Spanish patients with SJS/TEN. To gain more insight into the putative involvement of this cytokine in the pathogenesis of the disease, we have compared serum concentrations of acute and resolution samples, and we have explored the levels of the cytokine in blister fluids as well, when available. As the private receptor IL-15Rα has a central role for the presentation of the cytokine to responding cells, we have used quantitative RT-PCR (qPCR) to analyze the gene expression of *IL15RA* as well as of *IL15* in peripheral blood mononuclear cells (PBMC) and in affected skin from patients with this condition.

## 2. Patients and Methods

### 2.1. Patients and Patients’ Samples

The study included 53 validated SJS/TEN cases included in the Spanish registry PIEL*enRed* (22 males, 31 females; age range 2–93 years; median 45 years).

Clinical data, pictures and histological examinations for each patient were reviewed by an expert review committee blinded to drug exposures in order to establish a final diagnosis according to current classifications [1]. Overall, 31 definite, 18 probable and 4 possible cases were included, among which 10 were diagnosed as TEN, 19 as SJS and 24 were SJS/TEN overlap cases (Supplementary Materials Table S1).

Peripheral blood was drawn from patients during the acute disease (acute samples) or upon complete remission of the clinical symptoms (resolution samples). Peripheral blood mononuclear cells (PBMCs) were isolated by Ficoll/Hypaque (Amersham Biosciences, Amersham, UK) density gradient centrifugation of blood samples. Blister fluids were obtained from tense blisters by puncture aspiration into a syringe. Blister fluid cells (BFCs) were collected by centrifugation for further assays and blister fluids (BF) were harvested and frozen at −80 °C until analyzed.

PBMCs from anonymous healthy donors were obtained from the hospital blood bank.

For the isolation of peripheral blood and blister fluid monocytes, positive selection was performed by magnetic isolation using CD14 microbeads (Miltenyi Biotec GmbH, Bergisch Gladbach, Germany). Purity of positive fractions was at least 90%, as assessed by flow cytometry.

Skin biopsy specimens were taken from acute skin eruptions. Normal skin was obtained from patients with other conditions undergoing plastic reconstructive surgery, or as leftovers from other surgical procedures.

Biological samples obtained from each patient are shown in Supplementary Materials Table S2.

The study was conducted according to the Declaration of Helsinki principles and was approved by the Ethical Research Ethics Committee of the Príncipe de Asturias University Hospital, the coordinating center of the PIEL*enRed* Consortium, (code PER-MED-2010-01; date 28 July 2010). All patients or their legal representatives gave written informed consent for the collection of both personal data and biological samples.

### 2.2. IL-15 Protein Quantification by ELISA

IL-15 protein levels were quantified in serum and BF using a commercially available DuoSet ELISA kit (R&D Systems Europe, Abingdon, UK) according to the manufacturer’s instructions. A Synergy 4 microplate reader with Gen5™ analysis software (Biotek Instruments, Inc. Winooski, VT, USA) was used for automatic acquisition and the analysis of data.

### 2.3. Real-Time Quantitative RT-PCR

Total cellular RNA was isolated from PBMCs, BFCs or CD14+-isolated monocytes using the High Pure RNA Isolation Kit (Roche, Mannheim, Germany) according to the manufacturer’s instructions and 1 μg was reverse transcribed with random hexanucleotides and AMV reverse transcriptase (Roche) in a final volume of 20 μL for 1 h at 42 °C. For skin biopsies, RNA was extracted using TriReagent^®^ (Molecular Research Center, Inc. Cincinnati, OH, USA) according to the manufacturer’s instructions, and 500 ng was used for reverse transcription. 

The expression of human *IL15* and *IL15RA* genes was analyzed by PCR amplification of 1 μL aliquots of the resulting cDNAs in a total volume of 15 μL, in a Light Cycler (Roche) with FastStart DNA Master SYBR Green I (Roche). Standard curves for target mRNA expression were generated by amplifying 10-fold serial dilutions of known quantities of the specific PCR product. Quantification of target gene expression was obtained using Light Cycler system software version 4.05 (Roche, Mannheim, Germany). *B2M* was used as a housekeeping reference gene, except for experiments involving skin biopsies, in which *ARF5* was used as a reference. Relative units estimated from the quantification (Rq) represent the ratio between specific mRNA molecules/μL cDNA and molecules of *B2M* (or *ARF5*) mRNA/μL cDNA.

The primers used are shown in Table S3 in the Supplementary Materials.

### 2.4. Statistical Analysis

The statistical analysis was performed using GraphPad Prism v 9.0 software (San Diego, CA, USA). Data are shown as mean ± SD values, or median and interquartile ranges (IQR) as indicated in each figure. Data were analyzed with nonparametric *t*-tests for unpaired or paired samples. Correlation was assessed using Spearman correlation tests. A *p* value ≤ 0.05 was considered statistically significant.

## 3. Results

### 3.1. Study Subjects 

In this study, 53 patients with SJS/TEN were recruited by the Spanish consortium for the study of severe cutaneous adverse reactions PIELenRed. The distribution of phenotypes, severity scores and demographic characteristics is shown in Supplementary Materials Table S1. Suspected drugs and biological samples obtained from each patient are shown in Supplementary Materials Table S2. Twenty-two were males and thirty-one were females. The median age was 45 years (range 2–93 years). In total, 19 were diagnosed as SJS, 10 as TEN and 24 as SJS/TEN overlap. Only 4 patients died (7.5%), all of them within the SJS/TEN overlap diagnostic group. 

### 3.2. IL-15 Serum Levels in SJS/TEN

**Figure 1:**
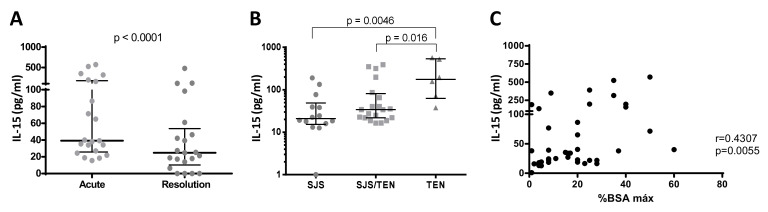
IL-15 protein levels in serum samples SJS/TEN patients. (**A**) Serum concentrations in acute and resolution samples. Median values and interquartile ranges (IQR) are indicated (N = 21; Wilcoxon *t*-test). (**B**) Serum IL-15 concentrations are shown in SJS (N = 14), SJS/TEN overlap (N = 20) and TEN (N = 6) patients. Median and IQR values are shown. Significantly higher values are found in TEN vs. SJS/TEN cases (Mann–Whitney U test). (**C**) Spearman correlation analysis of serum IL-15 concentrations and maximum body surface area (% BSA max) detached in each patient.

Serum acute samples were obtained from 40 cases at admission. In 21 of them, a second sample was obtained upon complete recovery (resolution samples). Serum IL-15 levels were significantly higher in the acute samples compared to the resolution samples from the same patients (Figure 1A), thus confirming the results of previous studies which suggested IL-15 involvement in the pathogenesis of the disease. Moreover, circulating levels of the cytokine were higher in TEN compared to SJS or SJS/TEN cases (Figure 1B) and positively correlated with the percentage of detached skin (Figure 1C).

A previous report showed a positive correlation between the severity of the reaction evaluated as the SCORTEN [26] and serum IL-15 concentrations in a large series of patients recruited by the international consortium RegiSCAR [24]. In our group of Spanish cases, a trend was found towards higher serum concentrations in patients with the higher scores (Supplementary Materials Figure S1A); however, we did not find an overall statistically significant correlation in our patients (Supplementary Materials Figure S1B).

### 3.3. IL-15 Protein Levels in Blister Fluid from SJS/TEN Patients 

**Figure 2:**
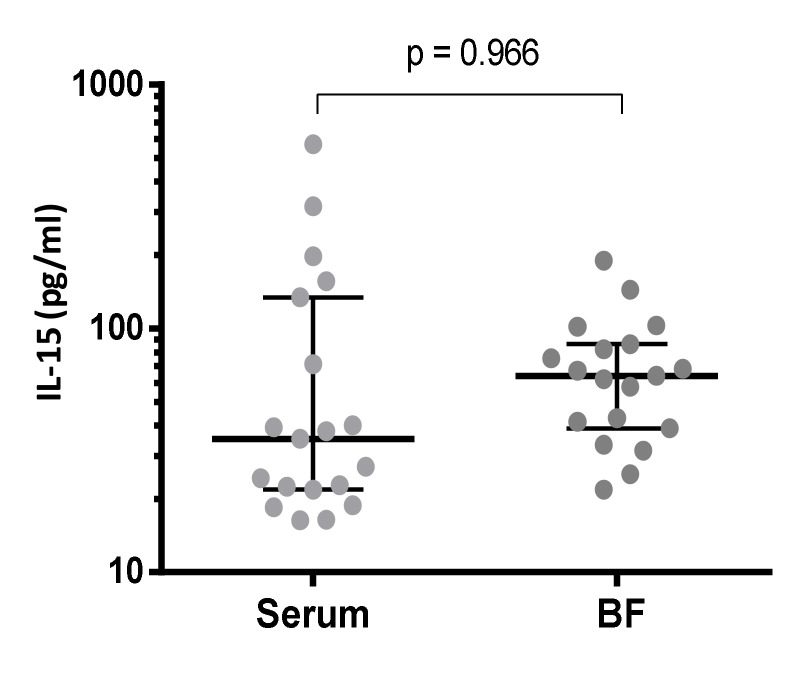
Comparison of acute serum and BF IL-15 concentrations in a set of 19 SJS/TEN patients. Median values and interquartile ranges (IQR) are shown (Wilcoxon test).

As the skin is the main target organ in SJS/TEN, IL-15 concentrations were also measured in blister fluid samples available from 19 cases and compared to circulating serum levels simultaneously obtained from the patients. Higher concentrations of IL-15 were detected in blister fluids, although the differences were not statistically significant (Figure 2). Contrary to the findings in serum, similar concentrations were observed in blister fluid samples when the patients were stratified according to the percent of detached BSA (Supplementary Materials Figure S2A), and no significant correlation was found with SCORTEN values (data not shown), although a trend to higher concentrations was found in those cases with higher scores (Supplementary Materials Figure S2B).

### 3.4. Gene Expression Levels of IL15 and IL15RA in PBMCs and BFCs from Patients with SJS/TEN

Given that IL-15Rα is critical for IL-15 signaling to immune cells, we analyzed *IL15RA* gene expression levels by quantitative RT-PCR (qPCR) analysis of mRNA transcripts in peripheral blood mononuclear cells (PBMCs) from patients with SJS/TEN and healthy donors. *IL15* gene expression was also analyzed in parallel.

**Figure 3:**
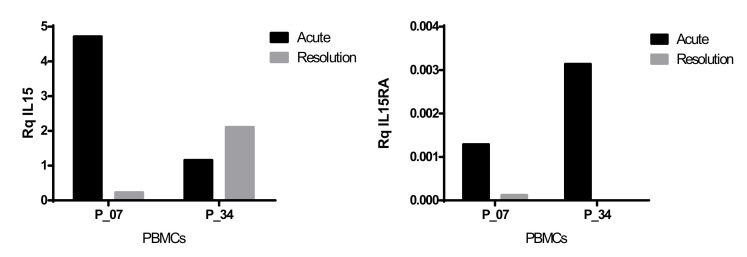
*IL15* and *IL15RA* gene expression analysis in PBMCs from acute and resolution samples in patients P07 and P34.

Median *IL15* gene expression levels were similar in PBMCs from SJS/TEN patients and healthy donors (Supplementary Materials Figure S3A), although a trend to higher mean values was found in SJS/TEN patients (Rq IL15 29.96 ± 79.0 vs. 8.3 ± 9.63). *IL15RA* transcripts were detected in about 70% of the individuals analyzed (11/18 SJS/TEN cases and in 13/19 healthy donors), with median *IL15RA* gene expression levels similar in patients and healthy donors (Supplementary Materials Figure S3B). However, mean values also tended to be higher in SJS/TEN cases than in healthy donors (Rq *IL15RA* 5 × 10^−3^ ± 1 × 10^−2^ vs. 2 × 10^−3^ ± 2 × 10^−3^). In this line, acute and resolution samples could be compared in two patients. A decrease in *IL15* expression was found upon the resolution of the disease in one of the cases analyzed, while both patients showed a marked decrease in *IL15RA* transcripts, with no expression detected in the second sample of case P34 (Figure 3).

**Figure 4:**
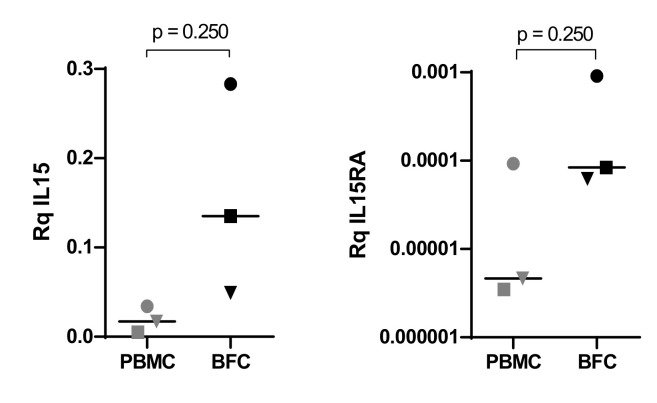
*IL15* and *IL15RA* gene expression analysis in acute PBMCs and BFCs from patients P03, P07 and P34 (Wilcoxon test).

*IL15* and *IL15RA* transcripts were also analyzed in BFC from three cases and compared to PBMCs from the same patients. As shown in Figure 4, the expression of both genes was higher in blister cells compared to peripheral blood mononuclear cells from the three patients analyzed.

### 3.5. Gene Expression Levels of IL15 and IL15RA in CD14+ Monocytes Isolated from PBMCs and BFCs from Patients with SJS/TEN

**Figure 5:**
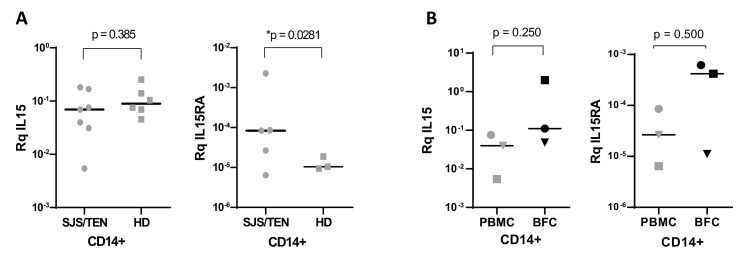
*IL15* and *IL15RA* gene expression analysis in CD14+ mononuclear cells. (**A**) *IL15* and *IL15RA* gene expression analysis in CD14+ peripheral blood monocytes isolated from SJS/TEN patients during the acute disease (N = 7) and from healthy donors (N = 6). (**B**) *IL15* and *IL15RA* gene expression analysis in CD14+ monocytes isolated from acute peripheral blood samples and BFCs of cases P03, P07 and P34. *p* = 0.0281

As monomyeloid cells are the main producers of IL-15 and responsible for transpresentation to effector lymphocytes, CD14+ monocytes were isolated from peripheral blood mononuclear cells and from blister fluid infiltrates and gene expression levels were analyzed. No differences were found in the expression of IL15 in CD14+-isolated monocytes from patients and control donors. IL15RA mRNA was detected in peripheral blood monocytes in five of seven SJS/TEN patients analyzed and in three of six healthy donors. Median values of IL15RA transcripts were significantly higher in monocytes from SJS/TEN cases (Figure 5A).

The expression of both genes was also investigated in CD14+ blister fluid cells (BFC) isolated from three patients and compared to peripheral blood CD14+ monocytes. A trend of higher expression levels of both cytokines was also found in the skin-infiltrating monocytes present in blister fluids compared to circulating CD14+ monocytes (Figure 5B).

### 3.6. Gene Expression Levels of IL15 and IL15RA in Skin Biopsies from Patients with SJS/TEN and Healthy Donors

**Figure 6:**
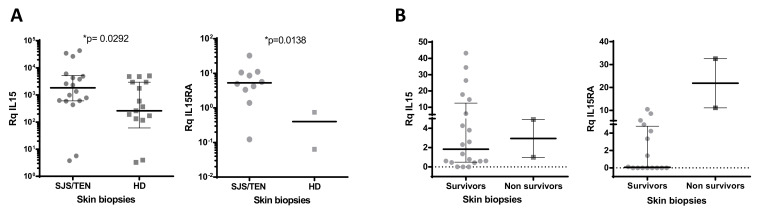
(**A**) *IL15* and *IL15RA* gene expression analysis in SJS/TEN-affected skin biopsies from SJS/TEN patients (N = 18) and in healthy skin (N = 17) * Mann–Whitney U test. (**B**) *IL15* and *IL15RA* gene expression analysis in SJS/TEN-affected skin from patients who did or did not survive the disease.

As many different cell lineages are capable of producing IL-15, including epidermal keratinocytes and fibroblasts, we sought to analyze and compare the expression of *IL15* and its private receptor *IL15RA* in skin biopsies available from 18 SJS/TEN cases and in the healthy skin from 17 donors. Significantly higher *IL15* gene expression levels were found in SJS/TEN samples (Figure 6A). *IL15RA* transcripts were detected in skin biopsies from 10 of the 18 cases analyzed, while in healthy skin it was only detected and in 2 cases, and very low amounts of mRNA were measured (Figure 6A).

No clear association was found with %BSA affected, although *IL15* expression tended to be higher in SJS/TEN overlap vs. SJS cases (Supplementary Materials Figure S4A), and a certain degree of correlation was found with total %BSA affected when only SJS and SJS/TEN overlap cases were considered for analysis (Supplementary Materials Figure S4C).

A correlation analysis was performed as well to explore the association of gene expression levels with the severity of the disease evaluated as the SCORTEN. The expression of *IL15* and *IL15RA* tended to be higher in cases with the higher scores, although the results did not reach statistical significance (r_(IL15)_ = 0.436, *p* = 0.061; r_(IL15RA)_ = 0.475, *p* = 0.171) (Supplementary Materials Figure S4E,F).

Nonetheless, skin biopsies were available from two of the patients who did not survive the disease. Interestingly, considerably higher levels of *IL15RA* transcripts were found in the skin of these two cases (Figure 6B). 

## 4. Discussion

Although IL-15 is a pleiotropic cytokine, one of its main actions is favoring the differentiation, the acquisition of effector functions, and the survival of cytotoxic lymphocytes, including effector memory cytotoxic T cells and NK cells, both involved in the induction of epidermal keratinocyte cell death in SJS/TEN.

Upon the initial screening of 28 soluble mediators, Su and collaborators found that IL-6, IL-8, TNF-α, granulysin and IL-15 were upregulated in patients with SJS/TEN at admission and selected for further analysis in 155 SJS/TEN cases. Granulysin and IL-15 were found to be related to disease severity as assessed by SCORTEN. Moreover, higher IL-15 levels were associated with mortality and in a small subset of cases, serial samples demonstrated a progressive increase in IL-15 in patients that did not survive, while levels were sustained or even decreased in survivors [24]. These findings led to the proposal of IL-15 as a biomarker and also as a putative therapeutic target [25].

In our study, we confirmed higher serum levels of IL-15 in the most severe cases as assessed by SCORTEN, although statistical significance was not reached. However, significantly higher concentrations were found in TEN cases compared to SJS or SJS/TEN and a significant correlation was found with the maximum % BSA detached in our patients. Blister fluid concentrations of IL-15 were also evaluated for comparative purposes and higher concentrations were found in blisters, although we could not establish a direct relationship between blister fluid IL-15 concentrations and SCORTEN or detached BSA, suggesting that the circulating levels of the cytokine would be more valuable as a prognostic or diagnostic biomarker of the disease. On the other hand, we could not evaluate the direct role of the cytokine as a predictor for mortality as serial serum samples or blister fluids were not available from non-survivors. Serial serum samples were analyzed from two patients (P48, P53) and serum IL-15 concentrations decreased over time and were lower at discharge in both cases (data not shown).

The production of IL-15 is regulated at the transcription, translation and secretion levels. In this sense, it is a unique cytokine because of its tightly regulated secretion and transpresentation, which are highly dependent on the availability of IL-15Rα, the IL-15 private alpha chain of the receptor. IL-15Rα chaperons IL-15 and helps its transportation from the endoplasmic reticulum to the cell membrane, where it remains mostly bound as a complex IL-15/IL-15Rα to be “transpresented” to responding cells [27]. Therefore, the amount of IL-15 accessible to activate responding lymphocytes expressing the IL-15Rβ/γc chains of the receptor is largely dependent on the IL-15Rα chain.

We have used quantitative RT-PCR analysis to explore the expression levels of *IL15* and *IL15RA* genes in mononuclear cells and the affected skin of SJS/TEN patients. Although no significant differences were found overall in PBMCs from cases and healthy donors, higher mean values in SJS/TEN cases and marked downregulation in resolution samples from two patients suggest that there is an induction of gene expression mainly affecting the *IL15RA* gene during the acute reaction. This upregulation was also observed when blister fluid cells were compared to PBMCs from the same patients (see Figure 4), pointing to the role of both genes in the pathogenesis of SJS/TEN. These findings were further supported by the analysis of gene expression in monocytes, which are the main producers of IL-15/IL-15Rα complexes among PBMCs. *IL15RA* gene expression was lower in circulating monocytes from healthy individuals. Moreover, both *IL15* and *IL15RA* genes tended to be expressed at higher levels in the skin-infiltrating monocytes found in blister fluids. It is possible that monocytes expressing higher levels of these genes are recruited to the skin or that the proinflammatory microenvironment is able to enhance or induce the expression of the cytokine and its receptor. As the IL-15/IL-15Rα complex can be shed from the cell membrane under inflammatory conditions, it is likely that the increased IL-15 protein concentrations found in biological fluids are also related to the increased expression of IL-15Rα in the same cells.

On the other hand, it has been described that fibroblasts and keratinocytes can also be a source of the cytokine [17,28]. The analysis of skin biopsies not only revealed significantly higher *IL15* transcripts, but also that the expression of *IL15RA* was mainly restricted to inflamed tissue, as it could not be detected in most of the healthy skin samples analyzed. Moreover, the highest amount of *IL15RA* transcripts was found in the affected tissue of the two patients who did not survive and from which skin samples were available, suggesting an important role of the stromal cells in the local lymphocyte activation in SJS/TEN through the expression of IL-15Rα, that in turn, would allow IL-15 signaling to responding cells in the affected organ.

Our study is limited by the small sample size regarding specific phenotypes (SJS, SJS/TEN overlap and TEN) and the availability of cell samples for further analysis involving leukocyte subpopulations, likely leading to a type 2 statistical error and precluding the identification of significant results. Nonetheless, our results confirm previous data, linking IL-15 with the pathogenesis of SJS/TEN and point to the induction of *IL15RA* gene expression as a limiting factor for IL-15 signaling functions in this disease. The results suggest that the effects of IL-15 in SJS/TEN patients may be highly dependent on the expression of its private receptor IL-15Rα in affected skin, and it is tempting to speculate that the cutaneous expression of IL-15Rα in SJS/TEN might provide an explanation for the tissue-specific immune cytotoxic response in this clinical entity, although additional research is needed to explore *IL15RA* gene and protein expression in other T cell-mediated cutaneous hypersensitivity reactions to confirm this hypothesis.

Further research is also needed to explore the role of IL-15 as a prognostic factor in SJS/TEN, particularly as a biomarker of the progression of the disease, and maybe for monitoring the response to immunomodulatory treatments. Available inhibitors of IL-15 signaling [29,30,31] could also be explored as therapeutic tools in SJS/TEN. At this point, it should be noted that TNF-α inhibitors have recently been proposed as a promising therapeutic option for SJS/TEN patients [32,33]. Previous studies had linked IL-15 production with TNF-α stimulation of secreting cells [34], and TNF-α-targeted therapies have been shown to reduce serum IL-15 levels in patients with rheumatic diseases [35,36]. In vitro and ex vivo investigations are warranted to explore the response of IL-15/IL-15Rα-producing cells to TNF-α inhibitors, as well as to other immunomodulatory approaches that could be offered to SJS/TEN patients.

## 5. Conclusions

Circulating serum IL-15 protein levels could be more valuable than blister fluid concentrations as a prognostic or diagnostic biomarker for SJS/TEN. The elevated serum and blister fluid concentrations of IL-15 in SJS/TEN might be dependent on an increased expression of its private receptor IL-15Rα, which is increased in peripheral blood monocytes as well as in skin-infiltrating mononuclear cells. Moreover, IL15RA expression is significantly induced in the affected skin of SJS/TEN patients, thus suggesting that stromal cells, such as keratinocytes and dermal fibroblasts, are contributors to IL-15 deleterious effects in this condition through IL-15 presentation to cytotoxic effector lymphocytes.

## Data Availability

All data are contained in the paper.

## Supplementary Materials

The following are available online at https://www.mdpi.com/article/10.3390/biomedicines10081868/s1, Table S1: Demographics and patients’ features. Table S2: Samples obtained from SJS/TEN cases and drugs involved. Table S3: Oligonucleotides used for quantitative RT-PCR analysis. Figure S1: Distribution of IL-15 serum concentrations in SJS/TEN cases according to SCORTEN and correlation analysis. Figure S2. IL-15 protein levels in blister fluid (BF) samples from SJS/TEN patients. Figure S3: IL15 and IL15RA gene expression in PBMCs from patients and healthy donors. Figure S4: IL-15 (and IL15RA gene expression analysis in skin biopsies from SJS/TEN patients.
