# Supplementary material for: IL-15/IL-15Rα in SJS/TEN: Relevant Expression of IL15 and IL15RA in Affected Skin
Source: Biomedicines. 2022 Aug 2;10(8):1868. doi: 10.3390/biomedicines10081868 (PMC9405300; doi:10.3390/biomedicines10081868)
Supplement: Supplementary file 1 [file biomedicines-10-01868-s001.zip › biomedicines-1771397-supplementary.pdf]

Supplementary Materials

Table S1. Demographics and patients' features

Patient ID	%BSA máx	Diagnostic	Sex	Age	SCORTEN	Outcome
P 01	1	SJS	Female	31	0	Discharged
P 02	7	SJS	Male	30	1	Discharged
P 03	4	SJS	Male	57	2	Discharged
P 04	1	SJS	Female	54	1	Discharged
P 05	8	SJS	Female	78	1	Discharged
P 06	75	TEN	Male	10	3	Discharged
P 07	60	TEN	Male	80	3	Discharged
P 08	50	TEN	Female	41	2	Discharged
P 09	1	SJS	Male	89	3	Discharged
P 10	28	Overlap	Male	32	1	Discharged
P 11	25	Overlap	Male	77	4	Discharged
P 12	15	Overlap	Female	50	2	Discharged
P 13	8	SJS	Male	34	1	Discharged
P 14	15	Overlap	Male	63	4	Discharged
P 15	22	Overlap	Male	49	2	Exitus
P 16	17	Overlap	Male	2	no data	Discharged
P 17	20	Overlap	Female	45	4	Discharged
P 18	20	Overlap	Female	39	1	Discharged
P 19	8	SJS	Female	40	1	Discharged
P 20	4	SJS	Male	93	3	Discharged
P 21	11	Overlap	Female	41	2	Discharged
P 22	50	TEN	Female	5	3	Discharged
P 23	25	Overlap	Female	85	3	Exitus
P 24	16	Overlap	Female	51	2	Discharged
P 25	35	TEN	Female	38	1	Discharged
P 26	20	Overlap	Female	76	2	Discharged
P 27	37	TEN	Male	4	3	Discharged
P 28	5	SJS	Female	27	0	Discharged
P 29	11	Overlap	Female	75	4	Exitus
P 30	28	Overlap	Female	67	3	Discharged
P 31	50	TEN	Female	27	1	Discharged
P 32	20	Overlap	Female	63	no data	Discharged
P 33	40	TEN	Male	53	2	Discharged
P 34	20	Overlap	Female	39	3	Discharged
P 35	35	TEN	Female	34	1	Discharged
P 36	12	Overlap	Male	82	4	Discharged
P 37	8	SJS	Male	35	2	Discharged
P 38	4	SJS	Female	54	2	Discharged
P 39	8	SJS	Male	17	0	Discharged
P 40	5	SJS	Female	43	3	Discharged
P 41	9	SJS	Male	41	2	Discharged
P 42	25	Overlap	MALE	62	5	Discharged
P 43	23	Overlap	MALE	74	2	Discharged
P 44	20	Overlap	Male	10	3	Discharged
P 45	20	Overlap	Male	17	3	Discharged
P 46	2	SJS	Female	48	2	Discharged
P 47	16	Overlap	Female	60	2	Discharged
P 48	20	Overlap	Female	78	2	Discharged
P 49	5	SJS	Female	68	3	Discharged
P 50	8	SJS	Female	45	1	Discharged
P 51	40	TEN	Female	31	2	Discharged
P 52	20	Overlap	Female	67	2	Exitus
P 53	8	SJS	Female	26	0	Discharged

Table S2. Samples obtained from SJS/TEN cases and drugs involved

Patient ID	Drug/s involved	SKIN biopsy	PBMC	Serum	BF	BFC
P_01	LTG		√	√		
P_02	Tetracyclins	√				
P_03	Ethambutol, Levetirazetam	√	√	√	√	
P_04	Undetermined	√		√		
P_05	Rifampicin	√				
P_06	LTG		√			
P_07	Vemurafenib	√	√	√	√	√
P_08	ALP	√	√	√	√	
P_09	Undetermined			√		
P_10	PHT		√	√	√	
P_11	ALP	√		√		
P_12	No drugs	√		√	√	
P_13	ALP	√	√	√	√	
P_14	PHT	√				
P_15	Undetermined	√				
P_16	Amoxicillin			√		
P_17	Mirtazapine	√		√		
P_18	Amoxicillin	√	√			
P_19	Lorazepam	√				
P_20	SMX		√	√		
P_21	Sulfasalazin			√		
P_22	Penicillin V			√	√	
P_23	Cefotaxime, Levofloxacin		√	√	√	
P_24	Undetermined		√	√	√	
P_25	Amoxicillin			√		
P_26	Ciprofloxacin, Cefazoline	√	√	√		
P_27	LTG			√	√	
P_28	Undetermined		√			
P_29	PHT		√			
P_30	Ciprofloxacin		√	√	√	
P_31	Undetermined		√			
P_32	No drugs		√			
P_33	Vancomycin, Cefotaxime			√	√	
P_34	Vancomycin		√	√	√	
P_35	Dexketoprofen	√	√	√	√	
P_36	Undetermined	√				
P_37	CBZ			√	√	
P_38	SMX			√		
P_39	LTG	√		√		
P_40	SMX			√		
P_41	Ibuprofen			√		
P_42	SMX			√		
P_43	Piperacillin/tazobactam, vancomycin			√	√	
P_44	LTG			√		
P_45	SMX			√	√	
P_46	Undetermined			√		
P_47	CBZ			√		
P_48	Undetermined			√		
P_49	No drugs			√		
P_50	Undetermined			√	√	
P_51	Undetermined			√		
P_52	Levetirazetam, Ipilimumab	√				
P_53	Mesalazine, Pantoprazol, Deflazacort			√	√	

ALP: Alopurinol; CBZ: Carbamazepine, LTG: Lamotrigine, PHT: Phenytoin, SMX: Sulfamethoxazole

TableS3. Oligonucleotides used for quantitative RT- PCR analysis

Primer Description	Sequence	Target Predicted product length (bp)
B2M forward primer	5’- CCA GCA GAG AAT GGA AAG TC- 3’	B2M 300 bp
B2M reverse primer	5’-´ GAT GCT GCT TAC ATG TCT CG 3’	
ARF5 forward primer	5´ ATT CTC ATG GTT GGC TTG GAT G 3´	ARF5 104bp
ARF5 reverse primer	5´ TTC TAC ATT GAA GCC TAT GGT TG 3	
IL15 forward primer	5’-ATGCTACTTTATATACGGAAA-3’	IL15 164 bp
IL15 reverse primer	´5´-AGAAGACAAACTGTTGTTTGC-3’	
IL15RA forward primer	5’- GGGAGTCCAGCGGTGTCCTGTG 3’	IL15RA 256 bp
IL15RA reverse primer	5’-GTC CGA TGG TGG TGG CAC TGG-3’	

Supplementary Figures

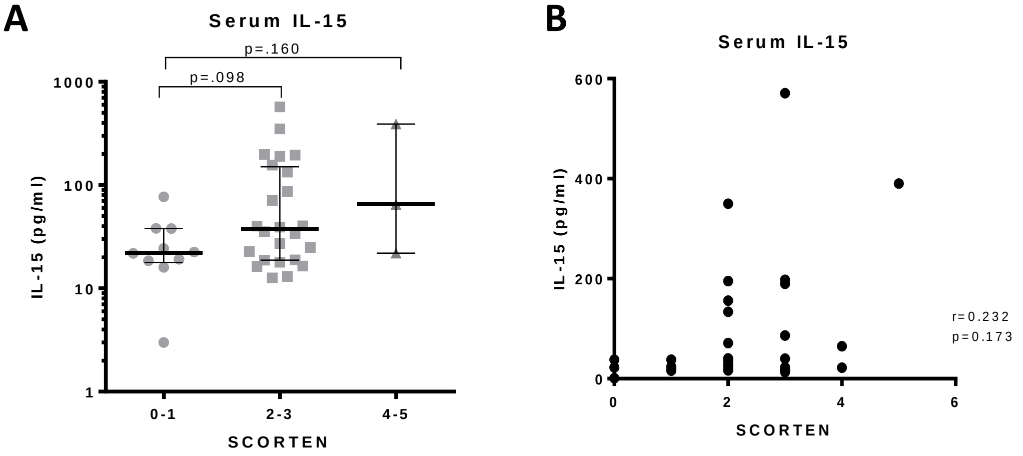

Figure S1. A. Distribution of IL-15 serum concentrations in SJS/TEN cases according to SCORTEN (Mann-Witheny U test was used to compared selected groups). **B.** Spearman correlation analysis of IL-15 serum concentrations and SCORTEN.

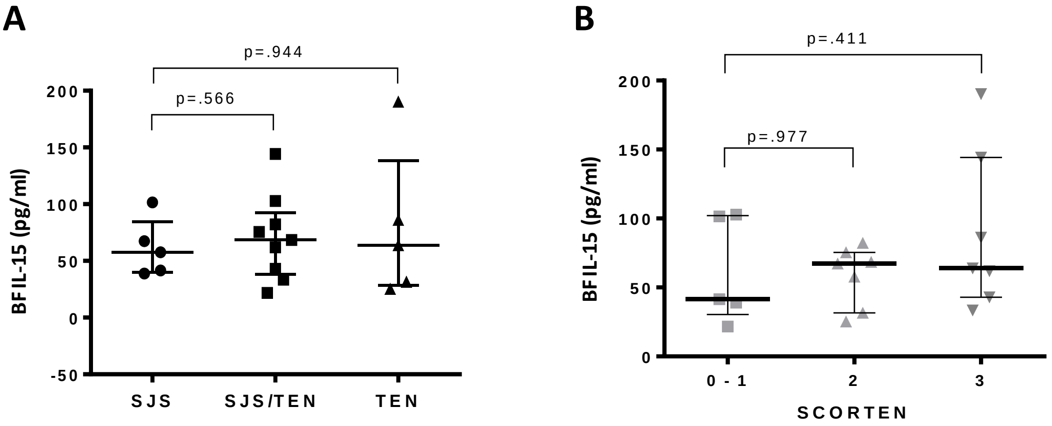

Figure S2. IL-15 protein levels in blister fluid (BF) samples from SJS/TEN patients. Median values and interquartile ranges (IQR) are shown. **A.** BF concentrations of IL-15 in SJS (N=5), SJS/TEN overlap (N=9) and TEN (N=5) patients (Mann-Whitney U test). **B.** BF concentration in SJS/TEN patients distributed according to the SCORTEN (Mann-Whitney U test).

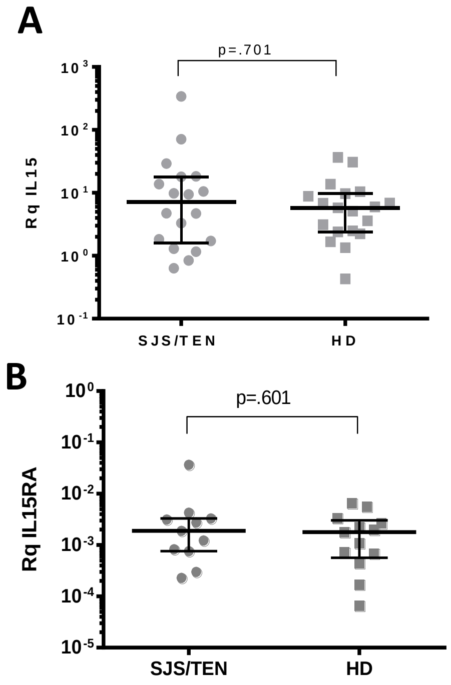

	SJS/TEN	HD
Number of values	18	19
Median Rq *IL15* (IQR)	7,091 (1.609-18.00)	5,789 (2.386-9.755)
**Mean Rq *IL15* ± SD**	**29,96 ± 79.00**	**8,308 ± 9.638**

	SJS/TEN	HD
Number of values	11	13
Median Rq *IL15RA* (IQR)	0.0019 ($7.5x10^{-4}$-$3.39x10^{-3}$)	0.00179 ($5.6x10^{-4}$-$3.0x10^{-3}$)
**Mean Rq *IL15RA* ± SD**	**0.005 ± 0.010**	**0.002± 0.002**

Figure S3. IL-15 (**A**) and IL15RA (**B**) gene expression analysis in PBMCs from SJS/TEN patients and healthy donors (HD). Median and IQR values are represented by horizontal bars. No significant differences were found in median expression values from cases and control donors (Mann-Whitney U test)

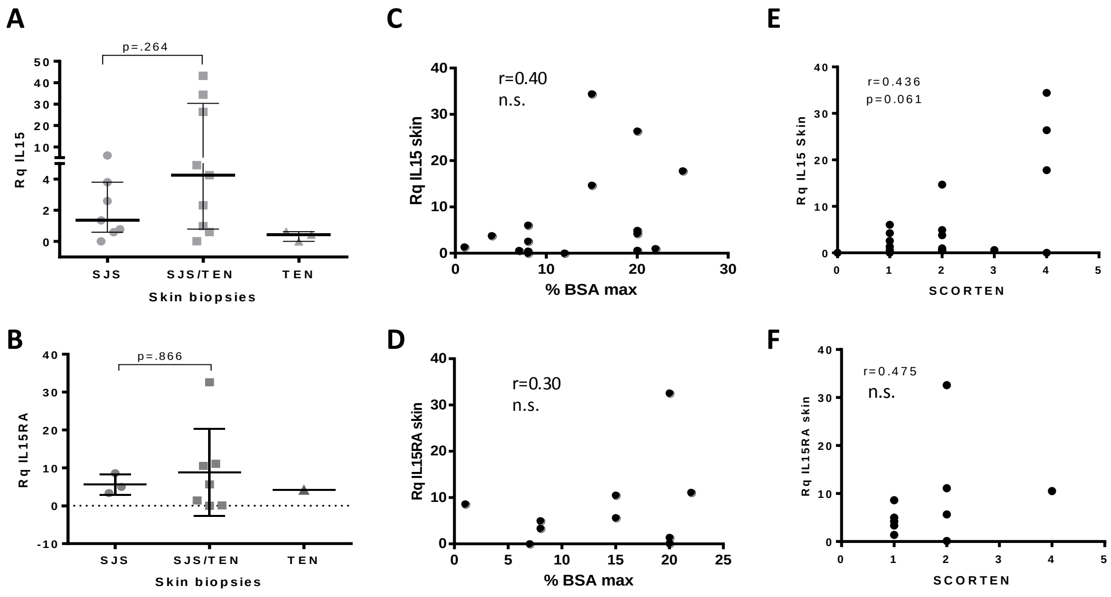

Figure S4. *IL-15* (upper panels) and *IL15RA* (lower panels) gene expression analysis in skin biopsies from SJS/TEN patients. **(A, B)** Gene expression levels are shown in SJS, SJS/TEN overlap and TEN cases Median and IQR values are represented by horizontal bars. No significant differences were found among the three groups analyzed (Mann—Whitney U test was used to compare SJS and SJS/TEN overlap cases). (**C, D)** Spearman correlation analysis of skin gene expression levels and maximum %BSA affected in SJS and SJS/TEN cases. (**E, F)** Spearman correlation analysis of skin gene expression levels and SCORTEN values in each patient.
